# Critical appraisal of clinical practice guidelines for depression in children and adolescents: A protocol for systematic review

**DOI:** 10.1097/MD.0000000000022384

**Published:** 2020-09-18

**Authors:** Li Du, Ya-Min Chen, Xiu Jin, Wei Yuan, Jian-Shu Wang

**Affiliations:** a The Third People's Hospital of Lanzhou city, Lanzhou, Gansu; b Evidence-Based Nursing Center, School of Nursing, Lanzhou University; c Department of General Surgery; d Department of Cardiovascular Medicine, the First Hospital of Lanzhou University; e Department of bone and soft tissue oncology, Gansu Provincial Cancer Hospital, Lanzhou, China.

**Keywords:** adolescents, children, clinical practice guidelines, depression, quality assessment

## Abstract

**Background:**

Depression as a clinically significant and growing public health issue is the third leading cause of disability. Adolescent-onset depression has been associated with psychiatric, anxiety disorders, suicidality, medical comorbidities, and an elevated risk of major depressive disorder episodes later in life. How to effectively identify, prevent, and treat depression has become one of the key points of discussion among medical institutions/departments. With the importance of depression being emphasized, countries/organizations around the world have developed guidelines for clinical practice related to depression for different groups of people to guide medical staff to implement scientific, effective, and standardized depression management. However, the quality of such guidelines is not yet clear, which is not conducive to the selection of medical staff and affects clinical application to a certain extent. This study aims to evaluate the rigor of the development of clinical practice guidelines (CPGs) for depression in children and adolescents and will identify, in these documents, the recommendations for depression in children and adolescents.

**Methods:**

Electronic databases and specific databases of CPGs will be searched. Study selection and data extraction will be performed independently by 2 reviewers. The AGREE II Instrument and RIGHT checklist will be used to assess the methodological quality and reporting quality of included CPGs about depression in children and adolescents. We will also analyze consistency and inconsistency of the recommendations in CPGs, including assessment, diagnosis, screening, treatment, and management. Bubble charts will be used to show the differences in methodological and reporting quality. Subgroup analysis will be conducted according to the result of evaluation. Excel and Endnote X9 will be used.

**Results:**

Using the search drafts of electronic databases, we included 6 CPGs. The results of our study will be published in a peer-reviewed journal.

**Conclusions:**

Our study will provide systematic evidence for existing CPGs for depression in children and adolescents and provide a guidance for CPGs users.

**Protocol Registration:**

INPLASY202080002.

## 1 Introduction

Depression as a clinically significant and growing public health issue is the third leading cause of disability, which surpassed only by diarrheal diseases and respiratory infections.^[1–3]^ Worthy of attention, depression is the leading cause of disability for young people, age 10 to 24 years.^[4]^ Approximately 300 million people were affected by this disorder in 2015. In 2016, approximately 5% of 12-year olds and 17% of 17-year olds reported experiencing a major depressive episode in the previous 12 months.^[5]^ Adolescence is the lifetime peak periods for onset of major depressive disorder (MDD).^[6]^ A previous study showed that early onset of depression has detrimental consequences on physical and mental development, and is associated to poorer academic, occupational, and social outcomes.^[7]^ Furthermore, adolescent-onset depression has been associated with psychiatric, anxiety disorders, suicidality, medical comorbidities, and an elevated risk of MDD episodes later in life.^[8]^ MDD is one of a leading global cause of burden,^[9]^ and the economic costs are considerable. For instance, the costs associated with the days lost of work due to depression and anxiety is estimated in US $ 1.15 trillion per year worldwide, and this amount is expected to increase 2-fold by 2030.^[10]^

The core clinical manifestations of depression symptoms include low mood, decreased interest or pleasure in most or all activities of the day, decreased motivation, increases or decreases in appetite and weight, insomnia or hypersomnia, psychomotor agitation or retardation, fatigue, cognitive impairments, such as memory deficit, and suicidal thoughts with or without suicidal plans or attempts.^[11]^ The current focus of treatment for people with confirmed MDD consists of antidepressants and psychotherapies. Wheresa antidepressants are typically more efficacious than placebo.^[12]^ Therefore, how to effectively identify, prevent, and treat depression has become one of the key points of discussion among medical institutions/departments.

The guideline recommendations were based on consensus in the guideline development group and relied on 3 sources: scientific evidence, experts’ opinions, and patient preferences.^[13]^ Clinical practice guidelines (CPGs) are the best guiding documents proposed after systematic evaluation of relevant scientific evidence and analysis of the pros and cons of various alternative intervention methods,^[14,15]^ which are one of the more authoritative tools that can help medical staff make decisions in specific clinical situations. With the elaboration of these documents, the concerns related to their quality increased.^[16–19]^ With the importance of depression being emphasized, countries/organizations around the world have formulated guidelines for clinical practice related to depression for different groups of people to guide medical staff to implement scientific, effective, and standardized depression management.^[20–24]^ However, the quality of such guidelines is not yet clear, and specific recommendations may vary depending on the target population, included evidence, and expert opinions, which is not conducive to the selection of medical staff and affects clinical application to a certain extent.^[25]^ This study aims to evaluate the rigor of the development of clinical practice guidelines for depression in children and adolescents and will identify, in these documents, the recommendations for depression in children and adolescents.

## 2 Methods

### 2.1 Study design

This systematic review of CPGs about depression in children and adolescents will include assessment, diagnosis, screening, treatment, and management. We will devise this study using the Appraisal of Guidelines for Research & Evaluation II (AGREE II) instrument^[26]^ and Instrument and Reporting Items for Practice Guidelines in Healthcare (RIGHT) checklist^[27]^ to evaluate the methodological and reporting quality of CPGs about depression in children and adolescents. At the same time, we will calculate the correlation between them using regression analysis and compare the recommendations in the guidelines.

### 2.2 Study registration

Our study has been registered in the International Platform of Registered Systematic Review and Meta-analysis Protocols (INPLASY) database (protocol number: INPLASY202080002, DOI: 10.37766/inplasy2020.8.0002). This protocol will adhere to Preferred Reporting Items for Systematic Reviews and Meta-Analyses (PRISMA-P).^[28]^

### 2.3 Data sources and search strategy

We will search PubMed, Web of Science, Cochrane Library, EMBASE.com, Chinese biomedical literature database (CBM). Specific databases for clinical guidelines will be searched, for example, the National Institute for Health and Clinical Excellence (NICE, https://www.nice.org.uk), Scottish Intercollegiate Guidelines Network (SIGN, https://www.sign.ac.uk), Guidelines International Network (GIN, https://www.g-i-n.net), Yimaitong website (http://www.medlive.cn), Chinese Medical Association (CMA, https://www.cma.org.cn). The MeSH search and text word search will be used with the terms related to the guideline, best practices, depression, depressive disorder, child or adolescent. To identify other possible guidelines, we will check the reference list of eligible studies, review studies, and secondary studies. For guidelines published only in summary or where important information is missing, we will try to search complete information by contacting the authors. A draft search strategy in PubMed is provided in Table 1.

**Table 1:**
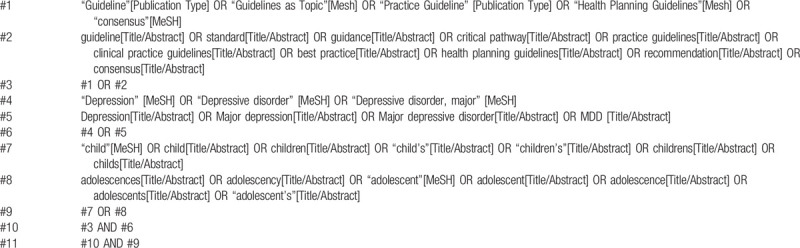
A draft search strategy in PubMed.

### 2.4 Eligibility criteria

#### 2.4.1 Inclusion criteria

Inclusion criteria are: issued in the form of guidelines or recommendations; mainly for depression in children and adolescents, which involves assessment, diagnosis, screening, treatment and management; language is limited to English and Chinese; if there is an updated relationship, the latest version is included.

#### 2.4.2 Exclusion criteria

Exclusion criteria are: the available version is incomplete or contains only a summary of the information; translation version based on the original; guidelines developed by individuals; consensus guideline, evidence summary, or algorithm.

### 2.5 Measured outcomes

We will conduct a systemic review of CPGs for depression in children and adolescents by evaluating methodological and reporting quality assessments and perform statistical analysis. At the same time, we will also analyze consistency and inconsistency of the recommendations in CPGs, including assessment, diagnosis, screening, treatment, and management.

### 2.6 Determination of eligibility

Records will be managed by EndNote X 8.0 (Thomson Reuters (Scientific) LLC Philadelphia, PA) software to exclude duplicates. At first, 2 reviewers (LD and YMC) will independently screen the titles and abstracts of the records to determine whether they met the inclusion criteria. Then, the same 2 reviewers find the full text of all potentially eligible studies and assess the eligibility of each study according to the inclusion criteria. Disagreements are resolved by discussion or by a third reviewer (JSW), or the whole group members will join the discussion.

### 2.7 Data extraction

Two main authors will independently collect data on study characteristics by using Microsoft Excel 2019 software to extract data from the included literature. A standard form is constructed and CPGs data is extracted, including: general information (title and subtitle, name of the first author, number of authors, year of publication, whether it is an updated version; type of CPGs [such as screening, diagnosis, treatment], country or region, organization, target user, target population); methodological information (types of studies included, evidence classification and evaluation criteria; recommendations formation method, recommendations classification standard, funding sources, update plan, peer review); recommendations (recommendations classification, contents of recommendations, strength of recommendation, quality of evidence).

### 2.8 Assessment of guideline quality

Reviewers (LD, YMC, XJ, and WY) who were trained to perform CPG appraisals using the AGREE II instrument and RIGHT checklist will conduct an independent review of the quality of each eligible CPG. Whenever a disagreement arises, we will resolve discordant evaluations by discussion to reach consensus and issue the final verdict. Finally, intraclass correlation coefficients (ICCs) will calculate to assess interrater reliability.^[29]^ ICCs will be calculated to assess inter-rater reliability and the measure of agreement between reviewers.^[29]^ The degree of agreement between 0.01 and 0.20 was deemed minor, 0.21 to 0.40 fair, 0.41 to 0.60 moderate, 0.61 to 0.80 substantial, and 0.81 to 1.00 very good.^[30,31]^

The AGREE II instrument consists of 23 items (each with specific reporting criteria) in 6 domains, including scope and purpose; stakeholder involvement; rigor of development; clarity of presentation; applicability; and editorial independence.^[26]^ Each item is rated on a 7-point Likert scale from strongly disagree to strongly agree (1–7, respectively) based on examples and instructions described in the AGREE II. The overall assessment included whether the CPG can be recommended for use in clinical practice. The consensus was reached according to the performance of partial item assessment and the global judgment by reviewers. Each CPG was classified as: “strongly recommended” for overall scores >60%, “recommended with modifications” for scores between 30% and 60%, and “not recommended” for scores <30%. The overall assessment was divided into 3 categories: recommended, recommended with modifications, and not recommended.^[32]^

The reporting quality of CPGs was appraised by the RIGHT checklist, which consists of 22 items: basic information (items 1–4), background (items 5–9), evidence (items 10–12), recommendations (items 13–15), review and quality assurance (items 16 and 17), funding, declaration, and management of interests (items 18 and 19), as well as information (items 20–22).^[27]^ Reviewers (YS, YG, JC, and LG) independently assessed the adherence of CPGs with the RIGHT checklist, each item is evaluated as “Yes,” “No,” and “Partial” according to its own reporting content.^[29]^

### 2.9 Data synthesis

For each CPG, the AGREE II score for each domain will be calculated as a percentage of the maximum possible score and standardized range, and the descriptive values included mean and standard deviation (SD). Reporting quality data plan to present as the number of RIGHT checklist items reported in each CPG, as well as the number of CPGs that reported individual RIGHT checklist items. STATA 14.0 software plan to be used for statistical analysis.

Descriptive analyses were conducted to summarize recommendations of the CPGs, including assessment, diagnosis, screening, treatment, and management, simultaneously. We will analyze the reasons for the inconsistency and consistency. Information on the strength of the recommendation and the level of evidence was extracted to determine the main gap between evidence. Bubble charts will be used to show the differences in methodological and reporting quality using Microsoft Excel 2019 (Microsoft Corp, Redmond, WA, www.microsoft.com). Subgroup analysis will be conducted according to the result of evaluation.

## 3 Results

### 3.1 Results of selected studies

**Figure 1:**
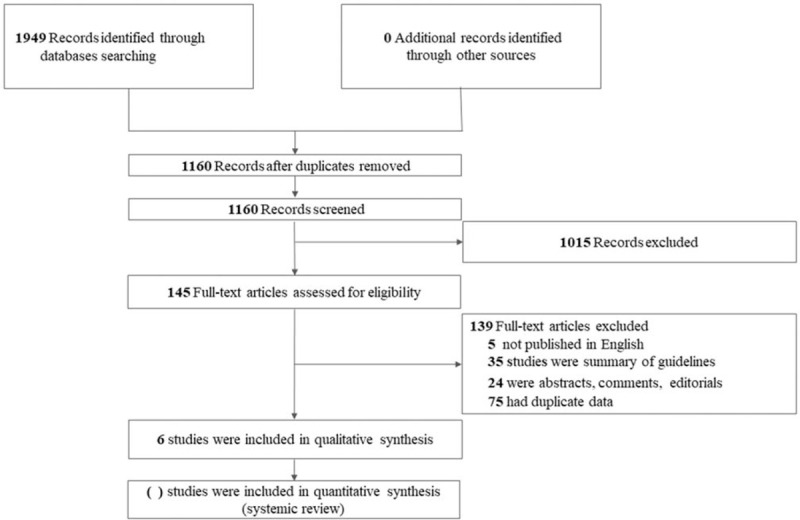
The flowchart of the screening process.

Using the search drafts of electronic databases and websites about guideline, 1949 records were identified, of which 789 duplicates were removed and 1160 records proceeded to title/abstract screening. After reviewing the titles and abstracts, 1015 records were excluded. Through full-text evaluation of the remaining 145 records, 139 studies were further excluded, we finally included 6 CPGs.^[20–24,33,34]^ The PRISMA flow chart of literature section is presented in Figure 1.

### 3.2 General characteristics of included studies

We extracted the basic characteristics of some of the included CPGs. We included 6 CPGs which were published from 2007 to 2019. The pages of guidelines were from 5 to 185. Only 1 reported the review situation as well as some of CPGs were funded by institute. We also extracted that whether the study reported search databases, whether to include appendix, whereas the information of evidence and recommendations. The details of characteristics of the included studies are summarized in Table 2.

**Table 2:**
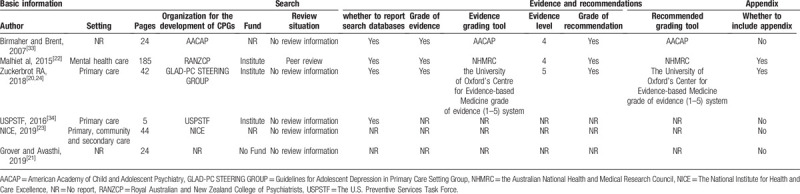
General characteristics of included studies.

## 4 Discussion

This study will identify CPGs of depression in children and adolescents, evaluate the methodological and reporting quality of included CPGs, and assess agreement among assessment, diagnosis, screening, treatment, and management of depression to inform consistent and inconsistent recommendations. The results of the study will show the advantages and disadvantages of the current depression CPGs in children and adolescents and provide a reference for users to select guidelines and benefit for future research. However, we only include English and Chinese guidelines, mainly for children and adolescents, which limits the promotion of conclusions. The results of the research will be submitted for publication in scientific journals, peer reviewed, and also published in national and international conferences.

## Author contributions

Li Du, Ya-Min Chen, Jian-Shu Wang conceived the study, developed the criteria, Li Du, Ya-Min Chen, Xiu Jin and Wei Yuan searched the literature, and analyzed the data. Li Du, Ya-Min Chen, Xiu Jin and Jian-Shu Wang wrote the protocol and revised the manuscript. All authors have read and approved the final manuscript.

**Conceptualization:** Jian-Shu Wang.

**Data curation:** Li Du, Ya-Min Chen, Xiu Jin, Wei Yuan, Jian-Shu Wang.

**Methodology:** Li Du, Ya-Min Chen, Jian-Shu Wang.

**Software:** Li Du, Ya-Min Chen, Xiu Jin, Wei Yuan, Jian-Shu Wang.

**Writing – original draft:** Li Du, Ya-Min Chen, Jian-Shu Wang.

**Writing – review & editing:** Li Du, Jian-Shu Wang.

## Abbreviations

Abbreviations: AACAP = American Academy of Child and Adolescent Psychiatry, AGREE II = appraisal of guidelines for research and evaluation II, CPGs = clinical practice guidelines, GLAD-PC STEERING GROUP = Guidelines for Adolescent Depression in Primary Care Setting Group, ICC = Intraclass correlation coefficient, INPLASY = the International Platform of Registered Systematic Review and Meta-analysis Protocols, MDD = major depressive disorder, NHMRC = the Australian National Health and Medical Research Council, NICE = The National Institute for Health and Care Excellence, PRISMA-P = Preferred Reporting Items for Systematic Reviews and Meta-Analyses, RANZCP = Royal Australian and New Zealand College of Psychiatrists, RIGHT = instrument and reporting items for practice guidelines in healthcare, SD = standard deviation, USPSTF = The U.S. Preventive Services Task Force.
